# Growth Performance of Local Chicken Breeds, a High-Performance Genotype and Their Crosses Fed with Regional Faba Beans to Replace Soy

**DOI:** 10.3390/ani10040702

**Published:** 2020-04-17

**Authors:** Tanja Nolte, Simon Jansen, Steffen Weigend, Daniel Moerlein, Ingrid Halle, Wolfgang Link, Jürgen Hummel, Henner Simianer, Ahmad Reza Sharifi

**Affiliations:** 1 Department of Animal Sciences, Animal Breeding and Genetics Group, University of Goettingen, 37075 Goettingen, Germany; hsimian@gwdg.de (H.S.); rsharif@gwdg.de (A.R.S.); 2 Center for Integrated Breeding Research, University of Goettingen, 37075 Goettingen, Germany; Steffen.Weigend@fli.de (S.W.); wlink@gwdg.de (W.L.); 3 Institute of Farm Animal Genetics, Friedrich-Loeffler-Institut, 31535 Neustadt, Germany; Simon.Jansen@fli.de; 4 Department of Animal Sciences, Quality of Animal Products, University of Goettingen, 37075 Goettingen, Germany; daniel.moerlein@uni-goettingen.de; 5 Institute of Animal Nutrition, Friedrich-Loeffler-Institut, 38116 Braunschweig, Germany; Ingrid.halle@fli.de; 6 Department of Crop Sciences, Division of Plant Breeding Methodology, University of Goettingen, 37075 Goettingen, Germany; 7 Department of Animal Sciences, Ruminant Nutrition, University of Goettingen, 37077 Goettingen, Germany; jhummel@gwdg.de

**Keywords:** carcass traits, faba bean, growth, local breeds, vicin

## Abstract

**Simple Summary:**

The culling of day-old male chicks and the ecological impact of high soy imports from overseas as animal feed are intensively discussed by the Western European agricultural sector and society. One possible approach to mitigate these problems could be the use of dual-purpose chickens for meat and egg production in combination with a predominant use of regionally grown protein plants. In the present study the suitability of six different chicken genotypes for fattening was evaluated while feeding them two different faba bean varieties. No adverse effects of the faba bean feeding on the performance and the health of the birds could be detected.

**Abstract:**

The faba bean (*Vicia faba* L.) is a native protein crop and considered a promising alternative to soybeans. Due to its anti-nutritive substances such as vicin and convicin (VC) its use in animal nutrition has been restricted. In the present study, two consecutive experiments were conducted to analyse the effects of feeding 20% faba beans, which differ in their VC content on fattening performance and slaughter traits of different chicken genotypes. In a first trial, purebred male chickens of the local breeds Bresse Gauloise and Vorwerkhuhn as well as of a high-performance White Rock line were tested. In a second trial, crossbreds of them were evaluated: Vorwerkhuhn × Bresse Gauloise, Vorwerkhuhn × White Rock, Bresse Gauloise × White Rock. Daily weight gain and feed intake were recorded until slaughter at approximately 2100 g. At slaughter the final live weight, carcass yield and the weights of the valuable parts (breasts and legs) were measured. For the genotypes studied, no adverse or undesirable effects of both VC−rich and VC−poor faba beans in the feedstuff were detected regarding body weight development, carcass quality, and fattening parameters. Furthermore, there was no indication that the birds’ health was impaired.

## 1. Introduction

Since the genetic correlation between growth rate and egg production is negative, in commercial poultry farming, predominantly crosses of specialized lines are used that have been selected either for a high laying performance or for a high growth rate and muscularity [1]. Therefore, in the layer industry the male chicks of layer hybrids are culled on the first day of life because of their low fattening performance. In Germany alone this accounts for more than 42 million chicks per year, given a sex ratio of 50% females and 50% males [2], which is problematic in terms of animal welfare, legal and ethical aspects, and social acceptance. In addition to methods of sex determination “in ovo”, which are currently under development, and extended laying periods [1,3], fattening of male layer chicks is considered to be a possibility for overcoming the killing of day-old chicks. In this context, the fattening of male chickens up to a maximum weight of 650 g, then called poussins [4], the fattening of the cocks of heavy layer hybrids or the use of dual-purpose-breeds [5,6] have been examined. All these studies have shown that the layer cockerels are clearly inferior to commercial broilers in terms of fattening performance and are consequently economically unprofitable under current production and market conditions. The use of local breeds could be an alternative solution to serve niche markets in a regional context. Previous to the industrialisation of poultry production in the middle of the last century, these breeds had been used for both, egg and meat production. Nowadays, local breeds are mostly kept by hobby breeders, and selection is focused more on phenotypic appearance than on growth or laying performance. Although the performance level of these local breeds is unfavourable compared to commercial layers and broilers [7,8], crosses of these breeds may perform better due to heterosis effects, as has already been shown by Götze and von Lengerken [8] with hybrids of different local breeds.

In chicken production, the high performance level of selected specialised broilers and layers, respectively, requires high protein content in animal feedstuff, which is usually achieved by using soybeans as a source of protein. About 2.5 million tons of soybeans are imported annually for feed production to Germany [9], which is seen critically by the German society due to the environmental impact of soy cultivation in South America and the high proportion of genetically modified soy [10,11]. Therefore, in Germany efforts are being made to reduce soy imports and to accelerate the cultivation of native protein crops [12].

Faba beans (*Vicia faba* L.) are considered a suitable alternative for poultry feed [13]. However, the endogenous glycosides vicin and convicin (together abbreviated as VC) are considered problematic (anti-nutritive), and thus limit the use of faba beans in human and animal nutrition. In humans, vicin and convicin are converted to the redox aglycones divicine and isouramil. These metabolites cause anaemia in people who are deficient in glucose-6-phosphate-dehydrogenase, the enzyme that physiologically detoxifies these substances. This genetic disposition in humans leads to the so-called favism [14]. It is known that a mutation of the VC− gene locus of the faba bean reduces the VC content in the faba bean seeds substantially [15], but there is currently no appropriate screening method for selection for low VC content available, and marker-assisted selection is presently only beginning. The difficulties to select for low VC content cause that only few low-VC varieties are available, for example the variety *Tiffany* [16].

The influence of faba beans in general and of VC in particular on the health and performance of chickens is not clear, and the results of different studies vary greatly. In laying hens, Olaboro et al. [17] showed VC to be responsible for reducing egg weights, which has been confirmed in other studies [18,19]. Experiments with commercial laying hens from Halle [20] fed with different concentrations of faba beans led to increased mortality and reduced laying performance. The recommended maximum levels of faba beans without adverse effects on the health of broilers vary between 125 g/kg [21] and 310 g/kg feed [22]. Causes for this wide variation can be found in the different experimental approaches of the studies cited.

Another main anti-nutritional factor besides VC is tannin. This substance has been shown to reduce the digestibility of protein as well as apparent metabolizable energy (AME_n_), with an additive effect of tannins with VC [23]. However, no effect of tannins on fattening performance was found [24,25]. In addition to the anti-nutritional factors, the amino-acid content of the faba bean has to be considered. Faba beans are deficient in sulphur-containing amino acids [13], which can be compensated by adding methionine to the diet in order to reduce performance losses [26]. Furthermore, not only the composition of the faba beans, but also the preparation of the feed has an influence on the acceptance by and growth performance of chickens. Ivarsson and Wall [27], as well as Gous [28], showed that the consumption of pelleted feed led to higher body weights compared to a mash-fed group. Similarly, Wilson and McNab [26] described a positive effect of autoclaving in comparison to the feeding of raw faba beans. There are two possible explanations for the positive effect of heat treatment on chicken performance. One is the destruction of heat-labile anti-nutritional factors, the other is a better availability of nutrients after heating.

Summarising the findings from all these studies, the safe amount of faba beans in the diet depends on many factors, that might occur together and interfere with each other in their effects on the animals. Furthermore, in contrast to commercial chicken lines, it is not known if and to what extent the feeding with faba beans influences the performance parameters of local chicken breeds.

The main objective of this study is to characterize the fattening performance of two purebred local breeds in comparison to a commercial line and crossbreds thereof, and the effect of feeding faba beans with different VC content on the performance traits of these genotypes.

## 2. Materials and Methods

### 2.1. Ethical Declaration

The experiment was performed in accordance with the German Animal Welfare Law and approved by the Lower Saxony State Office for Consumer Protection and Food Safety (LAVES) (reference number 33.9-42502-04-17/2622).

### 2.2. Stock and Husbandry

**Figure 1:**
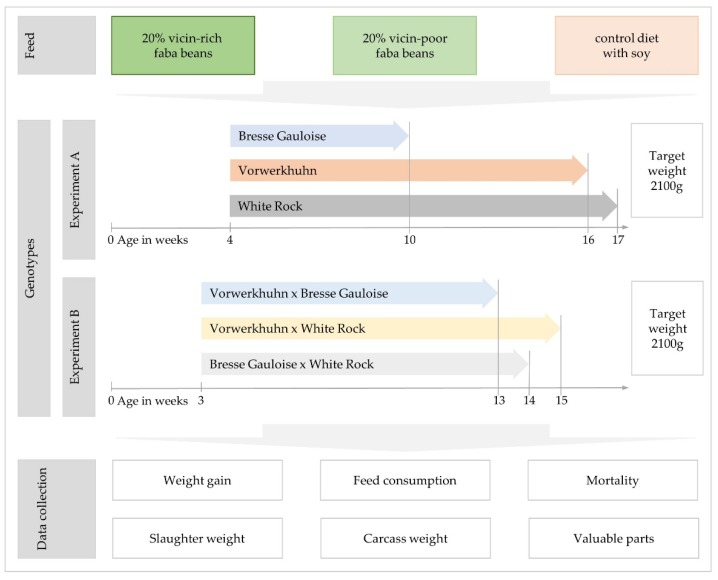
Schematic representation of the feeding treatments, genotypes, fattening period and collected data.

The experimental design applied in this research work is shown in Figure 1. According to the objectives of this study, two local breeds, the Vorwerkhuhn (VH) and Bresse Gauloise (BG), were included. The VH chicken breed is a German dual-purpose breed with an egg yield of up to 170 eggs per year and a cock weight of 2.2 kg at 16 weeks of age [29]. BG originates from the Bresse region in France and is marketed there as a delicacy with protected designation of origin (PDO). They have a laying performance of 240 eggs per year and the cocks weigh around 2.5 kg at 16 weeks of age [30]. For comparison with these local breeds, White Rock brown layer parent stocks (WR) from Lohmann Tierzucht GmbH were used. The cross of Vorwerkhuhn cocks and White Rock hens is known as Kollbecksmoorhuhn. This crossbreeding scheme was established in 2005 and has been used by breeders of the “Vorwerkerhaltungszucht” for more than 10 years to produce hybrid offspring for niche market production. These crosses have a higher productivity than the purebred VH chickens and support the conservation breeding activities [31].

For experiment A, day-old WR chicks were provided by Lohmann Tierzucht GmbH (Cuxhaven, Germany), while BG and VH chicks were reproduced from parent stocks at the Institute of Farm Animal Genetics of the Friedrich-Loeffler-Institut (Mariensee, Germany). After hatch, all chicks were marked with individual wing tags and reared for the first three weeks under the same conditions at the Institute of Animal Welfare and Animal Husbandry of the Friedrich-Loeffler-Institut in Celle, Germany. During the first three weeks of life, that is, before the beginning of the experiment, all chicks were fed the same commercial starter diet (11.4 MJ AMEn/kg DM, 180.0 g/kg crude protein, 26.1 g/kg crude fat, 37.5 g/kg crude fibre, 56.0 g/kg crude ash, 7.8 g/kg calcium, 4.7 g/kg phosphorous).

In the fourth week of life, 120 male chicks of BG and WR each and 94 male chicks of the VH breed were transferred to the Department of Animal Sciences of Goettingen University. The lower number of VH chicks was caused by low hatchability. The animals were divided into 12 pens per genotype (10 animals/pen for BG and WR, 7–8 animals/pen for VH) resulting in four replicates for each feeding treatment.

In experiment B, crossbreeds of the respective breeds (Vorwerkhuhn males × Bresse Gauloise females: VBG, Vorwerkhuhn males × White Rock females: VWR, and Bresse Gauloise males × White Rock females: BWR) were subjected to the same feeding regime as the purebred individuals. The feeding treatment in Goettingen started one week earlier than in experiment A, at the age of three weeks. Similar to the previous experiment, 120 animals of each cross were divided into 12 pens per genotype, again resulting in four replicates per feeding treatment.

The animals had ad libitum access to feed and water. The pens had a floor space of 2 × 1.5 m and were covered with wood shavings. They were equipped with perch, feeder and automatic cup-drinker. The room temperature was lowered from 22 °C at the beginning of the experiments to 20 °C from the 5th week of life. The light duration was 16 h per day.

### 2.3. Feeding Treatment

The animals of the two experiments were subjected to three different feeding treatments. While two diets contained faba beans as an alternative source of protein, the third diet was a soybean-based standard feed as control (soy). To investigate the influence of anti-nutritive substances on performance and health parameters, one of the experimental diets contained 20% of the VC-rich faba bean variety *Fuego* (VC+) and the other 20% of the VC-poor variety *Tiffany* (VC−). To meet the nutritional requirements of the chickens without soybean meal, 28.6% blue sweet lupines (*Lupinus angustifolius cv. Boruta*) and 10.5% peas (*Pisum sativum cv. Astronaute*) were also added to the two experimental diets. The feed was offered pelleted. The composition of the three different diets was calculated based on the recommendations of the German Society for Nutritional Physiology (GfE) and is presented in Table 1.

The VC content of the diets was measured with HPLC and the results showed that the VC content in the VC+ diets was 0.138% in experiment A and 0.136% in Experiment B. The VC content in the VC− diets was 0.022% and 0.016% in experiments A and B, respectively.

Dry matter, ash, crude protein, crude fibre, crude fat, neutral detergent fibre (NDF), starch and sugar were analysed according to VDLUFA (Association of German Agricultural Analytic and Research Institutes) methods [32] in the laboratory facilities of the Institute of Animal Nutrition of the Friedrich-Loeffler-Institut, Braunschweig, Germany. Tannin content was quantified with the AOAC Official Method 952.02 in the same laboratory. 

Fatty acids were analysed according to ASU L 13.00-27/3 + −46 and ISO 12966-3/4 and amino acids according to VDLUFA III 4.11.1 and 4.11.5 (both SYNLAB Analytics & Services GmbH, Jena, Germany). The amounts of saturated, monounsaturated and polyunsaturated fatty acids were calculated there based on the results of the fatty acid analysis. The nitrogen-corrected apparent metabolizable energy content was calculated on the basis of the chemical analysis with the WPSA formula [33].

### 2.4. Data Collection

To determine body weight development, all animals were weighed individually on a weekly basis over the experimental period. Feed consumption was measured weekly by weighing the amount of feed offered to the animals and the amount of remaining feed on a pen basis.

At the end of the experiment, a mixed sample of all feed bags from each of the diets was analysed in order to determine the chemical composition, the VC and Tannin content, fatty acid composition and the amino acids Methionine, Cysteine, Lysine and Threonine (Table 1).

Health status and mortality were checked daily. Mortality was recorded and the bodies of the deceased animals were examined for pathological changes and anatomical disorders.

The animals were slaughtered at a target weight of approximately 2100 g to assess fattening and slaughter performance. Therefore, the slaughter age of the breeds varied between 10 to 17 weeks of life (Figure 1). After 14 h of fasting, the animals were brought to the poultry slaughterhouse of the Department of Animal Sciences of Goettingen University.

Before slaughter, the weight of each animal was determined. The birds were electrically stunned and killed by neck cut. After scalding and plucking, the feet were removed and the carcasses eviscerated and rinsed. Until dissection on the following day, they were chilled at 1 °C. After storage for 24 h, the carcasses were weighed (without head, innards and feet) and dissected according to a standardized procedure. The weights of breast fillets (*M. pectoralis supf*., without skin) and legs (thigh + drumstick) were recorded. Carcass yield was calculated by determining the carcass weight as a percentage of live weight for each animal. The percentages of breast and leg were calculated as the portion of the respective part on the carcass weight.

Not all genotypes were slaughtered exactly at the planned target weight of 2100 g. The capacity of the laboratory facilities allowed only slaughtering one genotype per week, so that in case two genotypes in one experiment had a similar weight development, they had to be slaughtered in two consecutive weeks. This was the case with VH and WR in experiment A, and with VBG and BWR in experiment B. Limited laboratory capacity was also the reason the BG had to be slaughtered at an earlier time point and before reaching the target weight.

### 2.5. Statistics

Due to the different slaughter ages, the statistical analysis of the evaluated parameters was performed separately for each genotype. The statistical analysis of the weight gain data was first started by applying an initial model as a 4th-order polynomial growth function and then fitted using the backward selection approach. The initial model is represented as follows:

(1) $$y_{ijkl}=\mu +F_{i}+b_{s}W_{ij}+\overset{4}{\underset{v=1}{\sum }}b_{rv}{\left(A_{ij}\right)}^{v}+\overset{4}{\underset{v=1}{\sum }}b_{tv}F_{i}{\left(A_{ij}\right)}^{v}+p_{k}+e_{ijkl}$$

where $y_{ijkl}$ is the weekly weight, μ is the general mean, $F_{i}$ is the fixed effect of treatment (feeding group), $b_{s}$ is the fixed regression coefficient of the pre-treatment weight ($W_{ij}$),$\;b_{rv}$ are the fixed regression coefficients up to the fourth polynomial degree of age ($A_{ij}$), $b_{tv}$ are the fixed regression coefficients of the interaction between treatment and age, $p_{k}$ is the random effect of the pen and $e_{ijkl}$ is the random error. Using a backward selection, the non-significant regression coefficients of different polynomial degrees were removed from the initial model by applying F-statistics [34]. All statistical analyses were carried out using the procedure ‘mixed’ of the statistical program SAS (SAS 9.3., SAS Institute Inc., Cary, NC, USA). The final models for the respective genotypes are presented in Table 2. Least square means, which are adjusted means at average value of the considered covariates age and initial weight, were estimated by applying the least squares means (LSMEANS) statement. Significant differences between least square means were tested using Tukey-Kramer post-hoc tests by the PDIFF (*p*-value difference) option in the LSMEANS statement. Standard errors of least square means were calculated as described by Littell et al. [35].

For assessing the daily gain, the weight gain of the whole experimental period was divided by the number of fattening days and analysed using a linear mixed model with the feeding group as fixed and the pen as random effect. The analysis of daily feed intake (DFI) was performed using a similar statistical approach as for the growth. Therefore, the weekly feed intake per pen was transformed to daily feed intake per animal. The model is shown below, where $y_{ijkl}$ is the daily feed intake and the other variables as explained above:

(2) $$y_{ijkl}=\mu +F_{j}+\overset{4}{\underset{v=1}{\sum }}b_{rv}{\left(A_{ij}\right)}^{v}+\overset{4}{\underset{v=1}{\sum }}b_{tv}F_{i}{\left(A_{ij}\right)}^{v}+p_{k}+e_{ijkl}$$

The feed conversion ratio (FCR) was calculated by dividing the amount of feed consumed during the experiment through the weight gain over the same period. Statistical analysis was done using a linear mixed model with the treatment group as fixed and the pen as random effect. For the analysis of slaughtering parameters, the following linear mixed model was used:

(3) $$y_{ijkl}=\mu +F_{i}+b_{r}W_{j}+p_{k}+e_{ijkl}$$

where $y_{ijkl}$ is the respective parameter (carcass weight, carcass yield, breast and leg weight and percentage), μ is the overall mean, $F_{i}$ is the treatment (feeding group), $b_{r}$ is the regression coefficient of the pre-experimental weight ($W_{j}$), $p_{k}$ is the random effect of the pen and $e_{ijkl}$ is the random error.

For the time frame from 4 to 10 weeks of life, weight data of all six genotypes was available, so that a genotype comparison was done for this period. The weight at week 4 served as pre-treatment weight and was set as co-variable. The following model was used:

(4) $$y_{ijklm}=\mu +B_{i}+F_{j}+b_{s}W_{ijk}+b_{r}A_{ijk}+b_{r}A_{ijk}^{2}+B_{i}F_{j}+b_{r}B_{i}A_{ijk}+b_{r}B_{i}F_{j}A_{ijk}+p{\left(E\right)}_{l}+e_{ijklm}$$

where $y_{ijklm}$ is the weekly weight, μ is the general mean, $B_{i}$ is the fixed effect of genotype, $F_{j}$ is the fixed effect of treatment (feeding group), $b_{s}$ is the fixed regression coefficient of the pre-treatment weight ($W_{ijk}$), $b_{r}$ is the fixed regression coefficient of the age ($A_{ijk}$), $p{\left(E\right)}_{l}$ is the random effect of the experiment nested in pen and $e_{ijklm}$ is the random error. Additionally, for FCR a genotype comparison for the period from week 4 to 10 was performed. As fixed effects the genotype, the treatment group and their interaction were included in the model.

## 3. Results

### 3.1. Weight Gain

**Figure 2:**
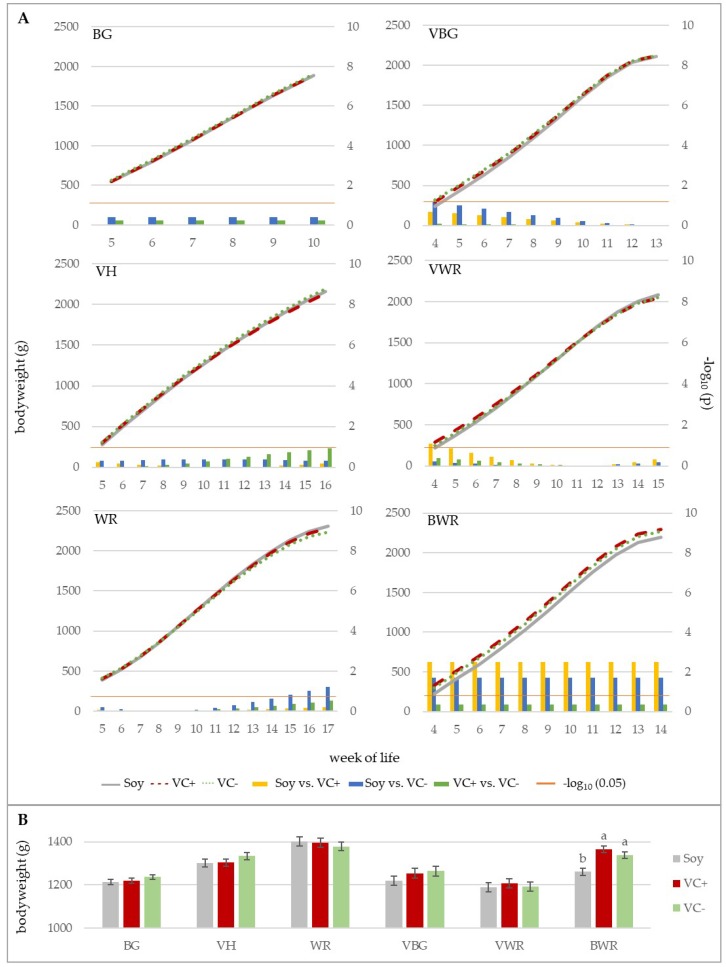
Effect of feeding treatment on bodyweight development within genotypes (**A**) and LSMEANS ± SE for the effect of feeding treatment within genotypes adjusted by the mean of age (**B**). In A, curves show bodyweight development for the respective feeding groups. The bar charts exhibit the differences between LS-means of the different combinations of feeding groups on a weekly base. The orange line represents the significance threshold of *p* = 0.05. Bars that cross this line imply significant differences between feeding groups in the respective week. In B bars with different letters within one genotype show significant differences (*p* < 0.05).

The development of bodyweight under the influence of feeding treatments is shown in Figure 2A. For experiment A, the animals of the BG breed were slaughtered in the 10th week of life. At this time, the live weight of the control group (soy) was 1883 g, while the weights of VC+ and VC− groups were 1888 g and 1905 g, respectively. Group VH was slaughtered in week 16 and the birds reached a final body weight of 2164 g (soy), 2139 g (VC+), and 2196 g (VC−). The WR chickens weighed 2308 g (soy), 2279 g (VC+), and 2233 g (VC−) when slaughtered in the 17th week of life.

For experiment B, the birds of the VBG group reached the target weight of 2100 g in the 13th week of life, as the final weights were 2114 g (soy), 2124 g (VC+), and 2122 g (VC−). Group BWR was slaughtered with 14 weeks and the weights were 2195 g, 2299 g and 2271 g in the soy, VC+ and VC− groups respectively. The VWR animals reached the slaughter weight in their 15th week of life. The respective weights for soy, VC+ and VC− were 2081 g, 2042 g and 2052 g.

The daily weight gains for all genotypes are presented in Table 3. The highest values were achieved by BG and the lowest by VH and WR. However, no significant effect of the feeding group on daily weight gain was found for the genotypes studied. The effect of the different variables on the body weight development of the different genotypes is presented in Table 2. The statistical model for the growth curves of the BG and WR fitted best with a third order polynomial degree of the age. For VH only a model with significant effects of the first and second order polynomial degree for the parameter age was selected. For the crossbreds VBG, VWR and BWR, a model with the fourth-order polynomial degree was found to be significant. While in BG and BWR no significant interactions between the fixed effect of the feeding treatment and the regression parameters of age could be detected, a significant effect of the interaction between the fixed effect of the feeding treatment and the linear regression term of age was observed in the genotypes VH, WR, VBG and VWR. Since the effect of the feeding treatment on weight development was only marginal, these interaction effects are negligible with respect to the extent of weight development (Figure 2A).

A significant effect of the feeding treatment is indicated for the crosses VBG and VWR (Table 2). However, this is neither reflected in the bodyweight development (Figure 2A), nor in the bodyweights adjusted by the mean of age (Figure 2B). The differences observed at the beginning of the experiment between the respective feeding groups decreased over time. These small differences were detected by the test statistics, but vanished when the whole period was taken into account. For the crossbred BWR, however, significant differences in weight development between the feeding treatments were found during the entire experiment. The soy-fed BWR group showed a significantly lower weight gain compared to the two faba bean groups. The difference between the groups was −105 g (Soy vs. VC+) and −77 g (Soy vs. VC−), respectively. This difference is also reflected in the mean bodyweights adjusted by the mean of age (Figure 2B).

**Figure 3:**
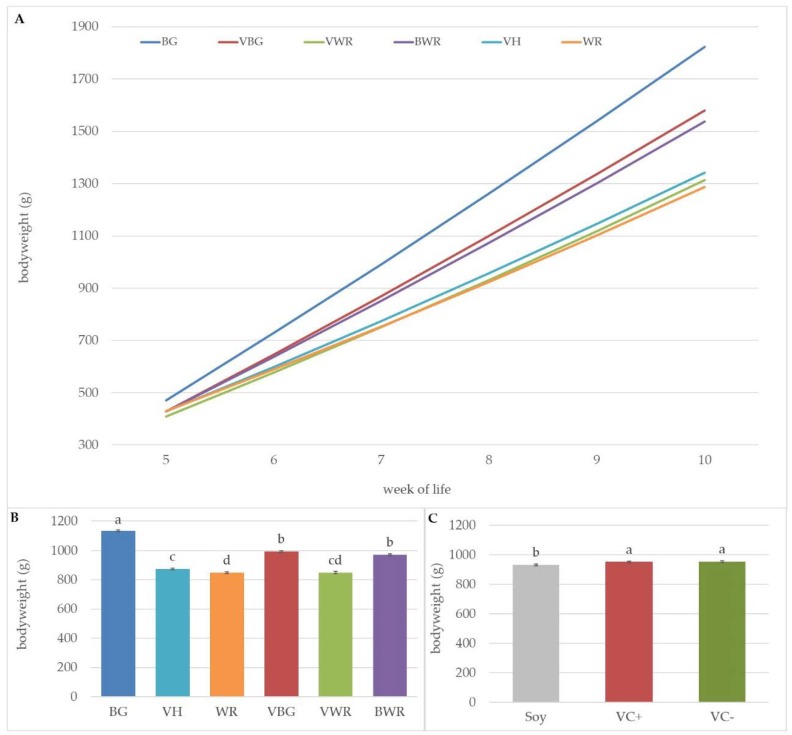
Comparison of the genotypes from week of life five to ten. (**A**) Effect of genotype on weight development. (**B**) LS-means ± SE for bodyweight of the respective genotypes adjusted by the mean of age. (**C**) LS-means ± SE for bodyweight of the different feeding groups adjusted by the mean of age. ^a,b,c,d^ Genotypes not sharing a letter differ at *p* < 0.05.

The comparison of the genotypes from week five to ten showed differences between the respective genotypes, which became clearer with increasing age (Figure 3A). In week ten the BG showed the significantly highest weights with 1823 g, followed by the two BG-crosses VBG and BWR (1580 g, 1536 g), which also differed significantly at this time point. The VH were with 1342 g significantly lighter than the BG and its crosses but heavier than the WR (1286 g). The VWR birds’ weights (1312 g) were in between VH and WR. The LS-means adjusted for the mean of age (Figure 3B) showed a similar picture but the difference between VBG and BWR was not statistically significant in this case.

Adjusted by the mean of age, the difference between the soybean and faba bean groups was statistically significant (Figure 3C), with the difference amounting to −19 g (soy vs. VC−) and −21 g (soy vs. VC+). Within the respective genotypes no significant differences between the feeding groups could be determined.

### 3.2. Mortality

The mortality in the present study was very low, of all 694 animals, only 5 animals died.

The mortality rate in experiment A across all breeds was 0.6%. For the VH breed the mortality rate was 1.1% and for the WR it was 0.8%. There were no losses observed for BG. Towards the end of the experiment, which overlapped with the beginning of sexual maturity, aggressive behaviour of the cocks among each other occurred, especially in the breed VH, which resulted in picking injuries.

The overall mortality at the end of the fattening period in experiment B amounted to 0.8%, which was similarly low as in in the purebred animals of experiment A. While no animal losses were observed in the BWR breed, the mortality rate for the VBG breed was 1.7% and for the VWR breed 0.8%. There was no influence of the feeding treatment on the mortality of the respective genotypes (*p* > 0.05).

### 3.3. Feed Intake and Efficiency

The feed efficiency for the different feeding groups and genotypes for the whole experimental period is presented in Table 4. No significant differences were found between the feeding groups of the respective breeds.

**Figure 4:**
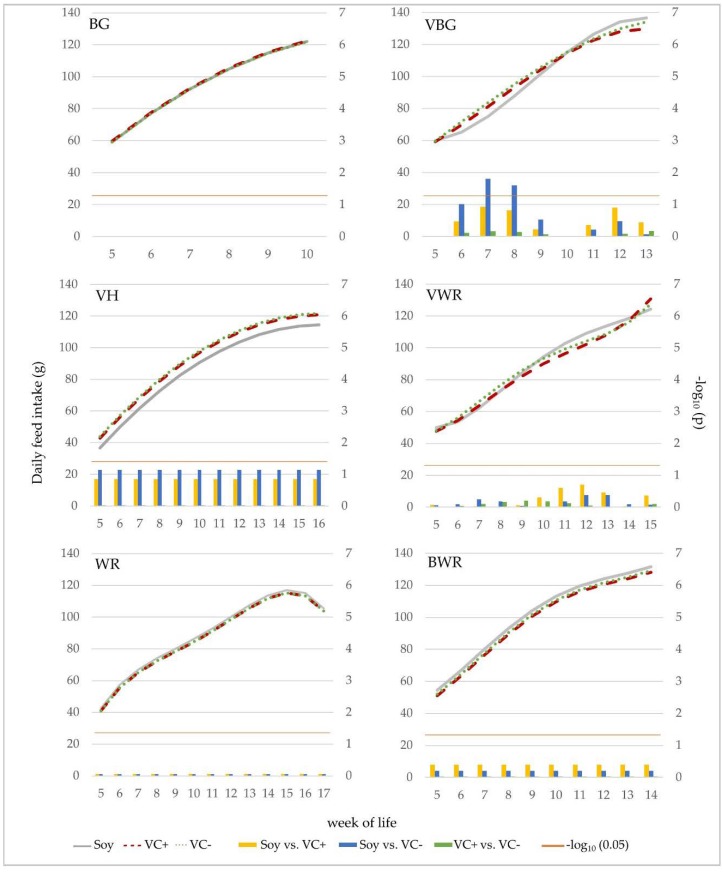
Effect of feeding treatment on daily feed intake (DFI) within genotypes. Curves show development of DFI for the respective feeding groups. The bar charts exhibit the differences between LS-means of the different combinations of feeding groups on a weekly base. The orange line represents the significance threshold of *p* = 0.05. Bars that cross this line imply significant differences between feeding groups in the respective week.

A comparison between genotypes was only possible for the time period from week 5 to 10, where data from all genotypes was available (Table 5). No statistically significant differences of the FCR were found between the feeding groups in general and within the respective genotypes as could also be seen in the separate analysis of the genotypes over the entire experimental period. In contrast, between the genotypes significant differences were detected. The BG showed the best feed efficiency (2.56 kg/kg) compared to all other genotypes. The VBG had a FCR of 2.76 kg/kg, which was significantly better than that of the VWR (2.91 kg/kg). The BWR, VH and WR were in between these two genotypes with FCRs of 2.74 kg/kg, 2.82 kg/kg and 2.84 kg/kg, respectively; differences were not significant. The LS-mean values of the daily feed intake (DFI) of the different feeding treatments of the genotypes are shown in Figure 4. The differences between the feeding groups within each genotype were rather small. Considering the whole experimental period, no significant differences in the mean DFI could be detected. Only in VBG chicks, the DFI differed significantly between the soy and VC− groups in week 7 and 8, hence the DFI of the soy group was reduced by 8 g per day.

The statistical model for the development of daily feed intake with age had the best fit at the polynomial degree of fourth order of age in the genotypes WR, VWR and BWR. For VBG it had to be reduced to the third order, and for BG and VH a model with only the first and second polynomial degree of age was significant. Significant interactions between the fixed effect of feeding treatment and the regression parameter of age were found only in VBG and VWR. For these two genotypes, the interaction was significant up to the polynomial degree of third order of the age.

### 3.4. Slaughtering Performance

The final weights and the measured parameters of fattening performance are shown in Table 6.

The yield, which represents the ratio of carcass to live animal weight, varied between 66.3% to 69.9% for the different genotypes. Within the genotypes the differences between diets were in general less than 1%. Only between the VC− and soy group of BG the difference was 1.2%, although not significantly different.

The breast yields were very similar in all six genotypes, too. The BG reached generally the highest values with the maximum of 13.3% in the soy group, while the VH and WR showed the lowest values with less than 11% in all feeding groups. The VWR yielded breast percentages of 11.3% (soy, VC+) and 11.2% (VC−). The breast yields of VBG and BWR have been slightly higher. In VBG the soy group had the highest breast yield with 11.9%, while in BWR the VC+ group performed best (12.0%). However, differences between the respective feeding groups were less than 0.5%.

With regard to leg yield, no fundamental differences were observed between the feeding groups of the six genotypes. The overall range was from 31.5% to 34.9% with maximum differences within genotypes of equal or less than 0.5%.

There were no significant differences in slaughtering parameters for the genotypes BG, VH, WR, VBG and VWR between feeding groups, while in contrast for the genotype BWR significant differences in the absolute weights of carcass, breast and leg were determined. The respective weights of the VC+ group were significantly higher than that of the soy group, which resulted in a difference of 99 g for carcass weight, 17.1 g for breast and 37.8 g for leg weight. The relative parameters carcass, breast and leg yield showed, as for the other genotypes, no significant differences.

The pre-experimental weights of the chicks affected the weights of carcass, breast and leg significantly in all six genotypes.

## 4. Discussion

The results of the present study suggest that feeding male birds of the six genotypes studied with 20% vicin-rich or vicin-poor faba beans as compared to a soybean-based diet had no negative effects on growth, feed efficiency and fattening performance.

This finding is in agreement with Farrel et al. [36], who recommended 20% faba beans as the maximum inclusion rate in broiler diets. With 24% faba beans, the authors noticed reduced weight gain in finishing broilers compared to the group with only 18% faba beans. Koivunen et al. [37] also observed lower bodyweight and reduced feed consumption with 24% faba beans in the diet compared to a soybean-based control. In contrast, another study found that even the feeding of 50% faba beans in the ration did not affect weight development, feed intake, dressing percentage and the weights of breasts and legs [38].

These aforementioned studies were conducted to assess the effects of faba beans in broiler feeding, but did not provide information on the VC content of the faba beans used.

Dal Bosco et al. [39] fed slow-growing broilers a ration with 16% faba beans and a VC content of 0.3%. This resulted in lower daily weight gain and reduced feed efficiency until day 60 and lower carcass weights in the faba bean group at slaughter. The carcass, breast and thigh yield were not affected by the feeding regime. The growth depression, that was observed during the early stage of development, is probably due to the higher susceptibility of younger animals towards the anti-nutritional factors of the faba bean such as VC or the higher requirements for essential amino acids at this age.

However, Laudadio et al. [22] found no differences in growth, feed intake and carcass traits of broilers fed 31% micronized faba beans with a VC content of 0.12% of dry matter.

In the present study, no influence of faba bean feeding on daily feed intake and feed efficiency was observed. In other studies even better feed efficiencies have been described when feeding 30% faba beans [36,40].

The mortality in the present study was rather low in both experiments A and B and no significant link to any of the feeding treatments was detected. These results are consistent with those from Laudadio et al. [22] and Gous [28], who did not report any influence of the faba bean feeding on the cases of death occurring during their experiments.

In addition to faba beans, which were with their differing VC contents the focus of the present study, the grain legumes blue sweet lupine and field pea were also used in the experimental diets to compose soybean meal free diets. Although the portion of lupines of 28.6% was clearly higher than recommended in the literature [41] and the total legume content was almost 60%, no disadvantage of the two faba bean groups in comparison to the control group was found in the present study. This may be an indication that the combination of different grain legume species allows a higher legume content in total without the anti-nutritional effects of the individual legumes. However, to prove this theory, further research is needed.

Due to the different ages at slaughter, a statistical comparison of the genotypes was only possible for a certain period of the fattening phase, while the carcass traits could only be compared descriptively. There are few studies available in the performance of BG and crosses thereof, and this is to the best of our knowledge the first study providing data regarding the other genotypes. Muth et al. [42] compared the fattening performance of BG males with that of ISA 657 broilers under organic conditions. In that study, BG reached a live bodyweight of 2570 g at an age of 84 days. The carcass yield was 69.1% and breast yield was 18.7%. Another study showed a yield of 66.6% and breast yield of 19% for BG at a slaughter age of 17 weeks [43]. These values are comparable with those reported by Lambertz et al. [44] for BG and their crosses with New Hampshire hens. Siekmann et al. [45] reported a breast yield of 12.7% in the commercial dual-purpose chicken Lohmann Dual after 9 weeks of fattening. However, in the studies referred to above both the superficial and profound breast muscles were taken together for determining the breast weight, while in the present study and the one of Siekmann et al. [45] only the superficial muscle (*M. pectoralis supf.*) was used, which is probably the reason for the lower breast yield.

Differences between the genotypes are visible in the growth curves. As the curves of VH, WR, VBG, VWR and BWR have already started to flatten, the different time points for the slaughter of these genotypes are reasonable. In contrast, the curve of BG still has a linear slope, indicating that higher weights would have been possible, because this breed is still growing fast.

When comparing the genotypes, the BG showed the highest growth rate, while the WR showed the lowest. The VH grew slightly faster than the WR, while the crossbreds each performed between their parental breeds. The difference between the purebreds is caused by the genetic background of these genotypes, which indicates the BG is a meat-type dual purpose breed, while the VH is a layer-type dual purpose breed and the WR is a parental layer genotype.

In the present study, the feed conversion ratio varied from 2.54 (BG, VC−) to 4.39 (VH, VC−) depending on the genotype and feeding group and on the time point of analysis. Due to technical reasons, feed wastage during the experiments could not be avoided completely and so the daily feed intake and FCR may be overestimated. Nevertheless, it has been reported in the literature that the feed efficiency of local breeds and layer males is inferior to that of broilers. The high specialization of broilers went along with a constant increase in feed efficiency, leading to feed conversion ratios (FCR) as low as 1.5 kg/kg under optimum conditions [46]. In contrast, Lichovníkova et al. [47] reported values of 3.1 for Ross Broilers and 3.8 for ISA brown cockerels after a fattening period of 90 days under free-range husbandry conditions. Local breeds as the Belgian Malines and the Schweizerhuhn required 2.55 and 2.73 kg of feed to produce 1 kg life weight [48] and Perella et al. [49] reported a FCR of 3.8 kg/kg for the Italian slow growing genotype Gaina. In the case of layer males, FCRs of up to 10.0 kg/kg have been reported, depending on the husbandry system and length of the fattening period [50].

To gain more information on the impact of faba bean feeding and the role of VC on certain chicken genotypes, further studies investigating the protein digestibility of the diets and the bursa weight and abdominal fat content of the chicken carcass would be of value.

## 5. Conclusions

Regardless of their VC content, the feeding of 20% faba beans during the fattening period did not affect the growth and fattening performance of the six examined genotypes, when compared to a standard soy diet. Therefore, the studied faba bean varieties, both VC rich and VC poor, do not appear to have adverse effects on animal mortality and growth performance for these chicken genotypes and could be an alternative to soybeans from this point of view.

Because of its high growth rate and good feed efficiency, the meat-type BG is the most suitable genotype for fattening among the tested ones. As a cross-breeding partner, BG shortens the fattening period and improves the feed efficiency of the layer-type genotypes VH and WR. However, because BG, VH and their crosses are dual-purpose chickens, the results of the present study need to be consolidated with performance data of the respective hens, which will be communicated in a separate publication.

## Figures and Tables

**Table 1:** Composition of diets and chemical analysis.

Item	Experiment A			Experiment B		
	Soy	VC+	VC−	Soy	VC+	VC−
Ingredient (%)						
Wheat	30.0	8.00	8.00	30.0	8.00	8.00
Corn	36.0	25.2	25.2	36.0	25.2	25.2
Soybean meal	24.4			24.4		
Blue sweet lupine cv. Boruta		28.6	28.6		28.6	28.6
Field pea cv. Astronaute		10.5	10.5		10.5	10.5
Field bean cv. Fuego		20.2			20.2	
Field bean cv. Tiffany			20.2			20.2
Grass meal	5.6	0.1	0.1	5.6	0.1	0.1
Soybean oil	0.2	2.7	2.7	0.2	2.7	2.7
Dicalcium phosphate	1.3	2.2	2.2	1.3	2.2	2.2
Calcium carbonate	1.0	0.7	0.7	1.0	0.7	0.7
Cattle salt (NaCl)	0.3	0.4	0.4	0.3	0.4	0.4
DL-Methionine	0.2	0.4	0.4	0.2	0.4	0.4
Broilerpremix ^1^	1.0	1.0	1.0	1.0	1.0	1.0
Chemical analysis						
Dry matter abs (%)	90.0	90.3	90.1	89.7	90.1	90.2
Ash (g/kg DM)	67.6	64.5	64.9	67.3	64.3	67.0
Crude protein (g/kg DM)	211.6	220.5	228.3	213.0	213.1	214.3
Crude fat (g/kg DM)	29.7	56.2	58.7	33.5	67.0	67.3
Crude fibre (g/kg DM)	43.8	60.4	68.5	45.2	72.0	74.0
NDF (g/kg DM)	123.3	124.2	132.7	148.5	128.9	136.6
Starch (g/kg DM)	472.2	423.7	402.6	480.2	416.6	413.1
Sugar (g/kg DM)	40.7	35.2	33.0	37.0	33.5	32.3
SFA (g/100g fat)	17.4	15.2	15.4	13.6	14.9	14.6
MUFA (g/100g fat)	22.7	26.6	26.6	24.4	26.7	26.8
PUFA (g/100g fat)	59.9	58.2	58.0	62.1	58.3	58.6
Methionine (%)	0.49	0.48	0.43	0.46	0.50	0.49
Cysteine (%)	0.30	0.27	0.29	0.29	0.29	0.30
Lysine (%)	0.97	1.01	1.07	0.90	1.08	1.09
Threonine (%)	0.71	0.66	0.69	0.68	0.69	0.69
Vicin (%)	0.005	0.095	0.016	0.00	0.094	0.013
Convicin (%)	0.003	0.043	0.006	0.00	0.042	0.003
VC (Vicin + Convicin; %)	0.008	0.138	0.022	0.00	0.136	0.016
Tannin (mg/g)	4.22	4.48	4.01	3.74	3.39	3.89
**Calculated energy content**						
AME_n_ (MJ/kg)	14.1	14.3	14.1	12.9	13.0	12.9

VC+: VC-rich faba bean diet, VC−: VC-poor faba bean diet, DL-Methionine: racemic mixture of dextrorotary and laevorotary Methionine, NDF: neutral detergent fibre, SFA: saturated fatty acids, MUFA: monounsaturated fatty acids, PUFA: polyunsaturated fatty acids, AME_n_: nitrogen-corrected apparent metabolizable energy; ^1^ vitamin-mineral premix provided per kg of diet: Fe, 32 mg; Cu, 12 mg; Zn, 80 mg; Mn, 100 mg; Se, 0.4 mg; I, 1.6 mg; Co, 0.64 mg; retinol, 3.6 mg; cholecalciferol, 0.088 mg; tocopherol, 40 mg; menadione, 4.5 mg; thiamine, 2.5 mg; riboflavin, 8 mg; pyridoxine, 6 mg; cobalamin, 32 µg; nicotinic acid, 45 mg; pantothenic acid, 15 mg; folic acid, 1.2 mg; biotin, 50 µg; choline chloride, 550 mg.

**Table 2:** Significance of sources of variation in the analysis of weight development.

Genotype	Effect	Type III Sum of Squares	
		F Statistic	*p* Value
BG	FG	0.98	0.4128
	Age	0.80	0.3721
	Age ^2^	5.42	0.0202
	Age ^3^	5.40	0.0205
	Start	465.46	<0.0001
VH	FG	1.11	0.3396
	Age	1490.51	<0.0001
	Age ^2^	197.13	<.0001
	Age × FG	2.73	0.0656
	Start	524.32	<0.0001
WR	FG	2.24	0.1406
	Age	54.46	<0.0001
	Age ^2^	339.34	<0.0001
	Age ^3^	405.57	<0.0001
	Age × FG	17.64	<0.0001
	Start	17.64	<0.0001
VBG	FG	4.23	0.0277
	Age	16.96	<0.0001
	Age ^2^	14.71	0.0001
	Age ^3^	23.08	<0.0001
	Age ^4^	32.76	<0.0001
	Age × FG	4.11	0.0166
	Start	255.28	<0.0001
VWR	FG	5.54	0.0161
	Age	8.71	0.0032
	Age ^2^	3.82	0.0509
	Age ^3^	10.43	0.0013
	Age ^4^	19.87	<0.0001
	Age × FG	18.35	<0.0001
	Start	439.35	<0.0001
BWR	FG	10.94	0.0034
	Age	8.75	0.0032
	Age ^2^	6.33	0.0120
	Age ^3^	11.72	0.0006
	Age ^4^	18.48	<0.0001
	Start	236.78	<0.0001

BG: Bresse Gauloise; VH: Vorwerkhuhn; WR: White Rock; VBG: VH male × BG female; VWR: VH male × WR female; BWR: BG male × WR female; FG: feeding group; Age: age in weeks; Age ^2,3,4^: age to the power of 2, 3, 4, Start: pre-experimental weight.

**Table 3:** Effect of feeding treatment on the daily weight gain (in g).

	Purebreds			Crossbreds		
	BG	VH	WR	VBG	VWR	BWR
**Soy**	34.9 ± 0.6	21.8 ± 0.6	22.1 ± 0.6	27.8 ± 0.5	23.2 ± 0.5	27.2 ± 0.6
**VC+**	35.3 ± 0.6	21.4 ± 0.6	22.1 ± 0.6	27.7 ± 0.5	22.5 ± 0.5	27.8 ± 0.6
**VC−**	35.7 ± 0.6	22.2 ± 0.6	21.2 ± 0.6	27.7 ± 0.5	23.0 ± 0.5	27.6 ± 0.6

Least square means ± SE. BG: Bresse Gauloise; VH: Vorwerkhuhn; WR: White Rock; VBG: VH male × BG female; VWR: VH male × WR female; BWR: BG male × WR female, VC+: VC-rich faba bean diet, VC−: VC-poor faba bean diet. Values in the same column with no superscript are not significantly different (*p* > 0.05).

**Table 4:** Least square means (±SE) for the effect of feeding treatment on feed conversion ratio (FCR, in kg/kg) and daily feed intake (DFI, in g).

		Purebreds			Crossbreds		
		BG	VH	WR	VBG	VWR	BWR
**FCR**	**Soy**	2.57 ± 0.03	3.93 ± 0.28	3.97 ± 0.09	3.42 ± 0.15	3.76 ± 0.13	3.75 ± 0.82
	**VC+**	2.58 ± 0.03	4.25 ± 0.28	4.04 ± 0.09	3.23 ± 0.15	3.76 ± 0.13	3.42 ± 0.82
	**VC−**	2.54 ± 0.03	4.39 ± 0.28	4.01 ± 0.09	3.56 ± 0.15	3.89 ± 0.13	3.53 ± 0.82
**DFI**	**Soy**	95.2 ± 1.6	86.9 ± 2.3	89.0 ± 2.5	95.6 ± 1.5	86.7 ± 2.6	96.1 ± 1.9
	**VC+**	95.7 ± 1.6	93.2 ± 2.3	87.2 ± 2.5	94.7 ± 1.6	84.2 ± 2.6	92.6 ± 1.9
	**VC−**	95.1 ± 1.6	94.2 ± 2.3	87.3 ± 2.5	96.1 ± 1.5	85.4 ± 2.6	93.6 ± 1.9

BG: Bresse Gauloise; VH: Vorwerkhuhn; WR: White Rock; VBG: VH male × BG female; VWR: VH male × WR female; BWR: BG male × WR female; FCR: feed conversion ratio; DFI: daily feed intake, VC+: VC-rich faba bean diet, VC−: VC-poor faba bean diet. Values in the same column with no superscript are not significantly different (*p* > 0.05).

**Table 5:** Least square means (±SE) for feed conversion ratio (FCR in kg/kg) from week four to ten by genotype · feeding treatment and by genotype.

	Purebreds			Crossbreds		
	BG	VH	WR	VBG	VWR	BWR
**Soy**	2.57 ± 0.06 ^a^	2.76 ± 0.06 ^a,b^	2.86 ± 0.06 ^b^	2.77 ± 0.06 ^a,b^	2.90 ± 0.06 ^b^	2.79 ± 0.06 ^a,b^
**VC+**	2.58 ± 0.06	2.90 ± 0.06	2.86 ± 0.06	2.75 ± 0.06	2.83 ± 0.06	2.70 ± 0.06
**VC−**	2.54 ± 0.06 ^a^	2.79 ± 0.06 ^a,b^	2.80 ± 0.06 ^a,b^	2.77 ± 0.06 ^a,b^	3.00 ± 0.06 ^b^	2.74 ± 0.06 ^a,b^
	2.56 ± 0.04 ^a^	2.82 ± 0.04 ^b,c^	2.84 ± 0.04 ^b,c^	2.76 ± 0.04 ^b,c^	2.91 ± 0.04 ^c^	2.74 ± 0.04 ^b^

BG: Bresse Gauloise; VH: Vorwerkhuhn; WR: White Rock; VBG: VH male × BG female; VWR: VH male × WR female; BWR: BG male × WR female, VC+: VC-rich faba bean diet, VC−: VC-poor faba bean diet. ^a,b,c^ Values in the same row with different superscripts are significantly different (*p* < 0.05).

**Table 6:** Effect of feeding treatment on live weight at slaughter and carcass traits.

	Feed	Live Weight	Carcass Weight	Yield	Breast Weight	Breast Percentage	Leg Weight	Leg Percentage
		(g)	(g)	(%)	(g)	(%)	(g)	(%)
BG	Soy	1883 ± 14	1272 ± 18	67.5 ± 0.5	170 ± 3	13.3 ± 0.1	405 ± 9	31.8 ± 0.3
	VC+	1888 ± 14	1262 ± 18	67.1 ± 0.5	163 ± 3	12.9 ± 0.1	398 ± 9	31.5 ± 0.3
	VC−	1905 ± 14	1269 ± 18	66.3 ± 0.5	165 ± 3	13.0 ± 0.1	406 ± 9	32.0 ± 0.3
VH	Soy	2164 ± 21	1442 ± 32	69.5 ± 0.3	155 ± 5	10.8 ± 0.2	478 ± 12	33.1 ± 0.4
	VC+	2139 ± 21	1407 ± 32	69.5 ± 0.3	152 ± 5	10.8 ± 0.2	465 ± 12	33.0 ± 0.4
	VC−	2196 ± 21	1448 ± 32	69.0 ± 0.3	156 ± 5	10.7 ± 0.2	478 ± 12	32.9 ± 0.4
WR	Soy	2308 ± 21	1571 ± 32	68.7 ± 0.5	169 ± 7	10.7 ± 0.3	538 ± 8	34.3 ± 0.4
	VC+	2279 ± 21	1573 ± 32	69.1 ± 0.5	170 ± 7	10.8 ± 0.3	538 ± 8	34.2 ± 0.4
	VC−	2233 ± 21	1518 ± 32	69.5 ± 0.5	161 ± 7	10.6 ± 0.3	519 ± 8	34.2 ± 0.4
VBG	Soy	2114 ± 25	1431 ± 25	68.7 ± 0.3	170 ± 4	11.9 ± 0.2	479 ± 10	33.5 ± 0.2
	VC+	2124 ± 25	1481 ± 22	68.5 ± 0.2	170 ± 4	11.5 ± 0.2	505 ± 9	33.9 ± 0.2
	VC−	2122 ± 25	1482 ± 25	68.8 ± 0.3	176 ± 4	11.8 ± 0.2	498 ± 10	33.6 ± 0.2
VWR	Soy	2081 ± 22	1431 ± 18	69.4 ± 0.3	162 ± 4	11.3 ± 0.2	492 ± 12	34.4 ± 0.3
	VC+	2042 ± 22	1407 ± 19	69.3 ± 0.3	159 ± 4	11.3 ± 0.2	487 ± 12	34.5 ± 0.3
	VC−	2052 ± 22	1412 ± 18	68.6 ± 0.3	158 ± 4	11.2 ± 0.2	488 ± 12	34.5 ± 0.3
BWR	Soy	2195 ± 20 ^b^	1505 ± 27 ^b^	68.9 ± 0.3	175 ± 4 ^b^	11.6 ± 0.2	521 ± 10 ^b^	34.6 ± 0.2
	VC+	2299 ± 20 ^a^	1604 ± 26 ^a^	69.9 ± 0.3	192 ± 4 ^a^	12.0 ± 0.2	559 ± 10 ^a^	34.9 ± 0.2
	VC−	2271 ± 20 ^a^	1573 ± 26 ^a,b^	69.5 ± 0.3	185 ± 4 ^a,b^	11.7 ± 0.2	547 ± 10 ^a,b^	34.7 ± 0.2

LS-means ± standard error. BG: Bresse Gauloise; VH: Vorwerkhuhn; WR: White Rock; VBG: VH male × BG female; VWR: VH male × WR female; BWR: BG male × WR female, VC+: VC-rich faba bean diet, VC−: VC-poor faba bean diet. ^a,b^ Means with different superscripts within one column and genotype show significant differences (*p* < 0.05).

## References

[1] Krautwald-Junghanns M.-E., Cramer K., Fischer B., Förster A., Galli R., Kremer F., Mapesa E.U., Meissner S., Preisinger R., Preusse G. (2018). Current Approaches to Avoid the Culling of Day-Old Male Chicks in the Layer Industry, with Special Reference to Spectroscopic Methods. Poult. Sci..

[2] German Federal Statistical Office (Destatis) (2019). Geschlüpfte Küken in Deutschland im Zeitraum 2008 bis 2018 nach ausgewählten Geflügelarten. Fachserie 3, Reihe 4.2.3.

[3] Hirt H. (2004). Töten männlicher Legeküken—Situationsanalyse Schweiz 2004. Org. eprints.

[4] Koenig M., Hahn G., Damme K. (2009). Nutzung männlicher Legehybriden als Stubenküken: Mastleistung und Schlachtkörperzusammensetzung. Mitteilungsblatt Fleischforsch. Kulmbach.

[5] Damme K., Ristic M. (2003). Fattening Performance, Meat Yield and Economic Aspects of Meat and Layer Type Hybrids. Worlds Poult. Sci. J..

[6] Kaufmann F., Nehrenhaus U., Andersson R., Wolfrum S., Heuwinkel H., Reents H.J., Wiesinger K., Hülsbergen K.-J. (2017). Duale Genetiken als Legehennen für die ökologische Legehennenhaltung. Ökologischen Landbau weiterdenken: Verantwortung übernehmen, Vertrauen stärken, Beiträge zur 14. Wissenschaftstagung Ökologischer Landbau, Freising-Weihenstephan, 7–10 March 2017.

[7] Hahn G., Deerberg F., Lange K. (1995). Mit Rassegeflügel Fleisch erzeugen?. DGS Mag..

[8] Götze S., von Lengerken G. (1997). “Alternativ-Ökologische” Eierproduktion: Lassen sich Wirtschaftsgeflügelrassen nutzen?. DGS Mag..

[9] German Federal Statistical Office (Destatis). https://www-genesis.destatis.de/genesis/online/data.

[10] Barona E., Ramankutty N., Hyman G., Coomes O.T. (2010). The Role of Pasture and Soybean in Deforestation of the Brazilian Amazon. Environ. Res. Lett..

[11] Hobbs J.E., Plunkett M.D. (1999). Genetically Modified Foods: Consumer Issues and the Role of Information Asymmetry. Can. J. Agric. Econ..

[12] German Federal Ministry of Food and Agriculture (BMEL) (2016). Ackerbohne, Erbse und Co. Die Eiweißpflanzenstrategie des Bundesministeriums für Ernährung und Landwirtschaft zur Förderung des Leguminosenanbaus in Deutschland.

[13] Food and Agriculture Organization of the United Nations (FAO) Faba Bean (*Vicia faba*). https://www.feedipedia.org/node/4926.

[14] Luzzatto L., Arese P. (2018). Favism and glucose-6-phosphate dehydrogenase deficiency. N. Engl. J. Med..

[15] Duc G., Sixdenier G., Lila M., Furtoss V., Huisman J., van der Poel A., Liener I. (1989). Search of Genetic Variability for Vicine and Convicine Content in *Vicia faba* L. A First Report of a Gene Which Codes for Nearly Zero-Vicine and Zero-Convicine Contents. Recent Advances of Research in Antinutritional Factors in Legume Seeds, Proceedings of the First International Workshop on ‘Antinutritional Factors (ANF) in Legume Seeds’, Wageningen, 23–25 November 1988.

[16] German Federal Plant Variety Office (2019). Beschreibende Sortenliste Getreide, Mais, Öl- und Faserpflanzen, Leguminosen, Rüben, Zwischenfrüchte 2019.

[17] Olaboro G., Marquardt R.R., Campbell L.D., Fröhlich A.A. (1981). Purification, Identification and Quantification of an Egg-weight-depressing Factor (Vicine) in Fababeans (*Vicia faba* L.). J. Sci. Food Agric..

[18] Lessire M., Gallo V., Prato M., Akide-Ndunge O., Mandili G., Marget P., Arese P., Duc G. (2017). Effects of Faba Beans with Different Concentrations of Vicine and Convicine on Egg Production, Egg Quality and Red Blood Cells in Laying Hens. Animal.

[19] Muduuli D.S., Marquardt R.R., Guenter W. (1982). Effect of Dietary Vicine and Vitamin E Supplementation on the Productive Performance of Growing and Laying Chickens. Br. J. Nutr..

[20] Halle I. (2005). Einfluss gestaffelter Anteile von je zwei Erbsen- und Ackerbohnensorten im Legehennenfutter auf die Leistungsmerkmale. Landbauforsch. Völkenrode.

[21] Rubio L.A., Brenes A., Castaño M. (1990). The Utilization of Raw and Autoclaved Faba Beans (*Vicia faba* L., Var. Minor) and Faba Bean Fractions in Diets for Growing Broiler Chickens. Br. J. Nutr..

[22] Laudadio V., Ceci E., Tufarelli V. (2011). Productive Traits and Meat Fatty Acid Profile of Broiler Chickens Fed Diets Containing Micronized Fava Beans (*Vicia faba* L. Var. Minor) as the Main Protein Source. J. Appl. Poult. Res..

[23] Vilariño M., Métayer J.P., Crépon K., Duc G. (2009). Effects of Varying Vicine, Convicine and Tannin Contents of Faba Bean Seeds (*Vicia faba* L.) on Nutritional Values for Broiler Chicken. Anim. Feed Sci. Technol..

[24] Abel H., Gerken M. (2004). Ackerbohnen als Futterkomponente des ökologischen Landbaus für Masthühner-Elterntiere und verschiedene Mastbroilerherkünfte. Org. eprints.

[25] Woyengo T.A., Nyachoti C.M. (2012). Ileal Digestibility of Amino Acids for Zero-Tannin Faba Bean (*Vicia faba* L.) Fed to Broiler Chicks. Poult. Sci..

[26] Wilson B.J., McNab J.M. (1972). The Effect of Autoclaving and Methionine Supplementation on the Growth of Chicks given Diets Containing Field Beans (*Vicia faba* L.). Br. Poult. Sci..

[27] Ivarsson E., Wall H. (2017). Effects of Toasting, Inclusion Levels and Different Enzyme Supplementations of Faba Beans on Growth Performance of Broiler Chickens. J. Appl. Poult. Res..

[28] Gous R.M. (2011). Evaluation of Dehulled Faba Bean (*Vicia faba* Cv. Fiord) as a Protein Source for Broilers. S. Afr. J. Anim. Sci..

[29] Initiative zur Erhaltung alter Geflügelrassen e.V. Leistungsdaten Vorwerkhuhn. https://erhaltungszucht-gefluegel.de/index.php?id=39.

[30] Schultz S. Gefluegel-Erhaltungszucht.de—Die Bresse-Gauloise. http://www.gefluegel-erhaltungszucht.de/bresse-gauloise/.

[31] Weigend S., Stricker K., Röhrssen F.G. (2009). Establishing a Conservation Flock for “Vorwerkhuhn” Chicken Breed—a Case Study of in-Situ Conservation of Local Chicken Breeds in Germany. Anim. Genet. Ressources Inf..

[32] Association of German Agricultural Analytic and Research Institutes (VDLUFA) (1976). Handbuch der landwirtschaftlichen Versuchs- und Untersuchungsmethodik (VDLUFA-Methodenbuch), Bd. III. Die chemische Untersuchung von Futtermitteln.

[33] World Poultry Science Associaton (WPSA) (1984). The Prediction of Apparent Metabolizable Energy Values for Poultry in Compound Feeds. Worlds Poult. Sci. J..

[34] Köhn F., Sharifi A.R., Simianer H. (2007). Modeling the Growth of the Goettingen Minipig. J. Anim. Sci..

[35] Littel R.C., Milliken G.A., Stroup W.W., Wolfinger R.D. (2000). SAS System for Mixed Models.

[36] Farrell D.J., Perez-Maldonado R.A., Mannion P.F. (1999). Optimum Inclusion of Field Peas, Faba Beans, Chick Peas and Sweet Lupins in Poultry Diets. II. Broiler Experiments. Br. Poult. Sci..

[37] Koivunen E., Tuunainen P., Rossow L., Valaja J. (2013). Nutritive Value of Faba Bean (*Vicia faba* L.) Diets for Broilers. Proceedings of the 19th European Symposium on Poultry Nutriton.

[38] Moschini M., Masoero F., Prandini A., Fusconi G., Morlacchini M., Piva G. (2005). Raw Pea (Pisum Sativum), Raw Faba Bean (*Vicia faba* var. minor) and Raw Lupin (*Lupinus albus* var. multitalia) as Alternative Protein Sources in Broiler Diets. Ital. J. Anim. Sci..

[39] Dal Bosco A., Ruggeri S., Mattioli S., Mugnai C., Sirri F., Castellini C. (2013). Effect of Faba Bean (*Vicia faba* Var. Minor) Inclusion in Starter and Growing Diet on Performance, Carcass and Meat Characteristics of Organic Slow-Growing Chickens. Ital. J. Anim. Sci..

[40] Usayran N.N., Sha’ar H., Barbour G.W., Yau S.K., Maalouf F., Farran M.T. (2014). Nutritional Value, Performance, Carcass Quality, Visceral Organ Size, and Blood Clinical Chemistry of Broiler Chicks Fed 30% Tannin-Free Fava Bean Diets. Poult. Sci..

[41] Jeroch H., Lipiec A., Abel H., Zentek J., Grela E.R., Bellof G. (2016). Körnerleguminosen als Futter- und Nahrungsmittel.

[42] Muth P.C., Ghaziani S., Klaiber I., Valle Zárate A. (2018). Are Carcass and Meat Quality of Male Dual-Purpose Chickens Competitive Compared to Slow-Growing Broilers Reared under a Welfare-Enhanced Organic System?. Org. Agric..

[43] Pinent T., Reis L., König J.D.S. (2015). Vergleich von Merkmalen der Mast- und Schlachtleistung sowie von Überlebensraten bedrohter Hühnerrassen in einem standardisierten Versuchsdesign. Züchtungskunde.

[44] Lambertz C., Wuthijaree K., Gauly M. (2018). Performance, Behavior, and Health of Male Broilers and Laying Hens of 2 Dual-Purpose Chicken Genotypes. Poult. Sci..

[45] Siekmann L., Meier-Dinkel L., Janisch S., Altmann B., Kaltwasser C., Sürie C., Krischek C. (2018). Carcass Quality, Meat Quality and Sensory Properties of the Dual-Purpose Chicken Lohmann Dual. Foods.

[46] Aviagen Group (2017). Ross 308 AP Broiler: Performance Objectives.

[47] Lichovníková M., Jandásek J., Jürzl M., Dračková E. (2009). The Meat Quality of Layer Males from Free Range in Comparison with Fast Growing Chickens. Czech J. Anim. Sci..

[48] Mueller S., Kreuzer M., Siegrist M., Mannale K., Messikommer R.E., Gangnat I.D.M. (2018). Carcass and Meat Quality of Dual-Purpose Chickens (Lohmann Dual, Belgian Malines, Schweizerhuhn) in Comparison to Broiler and Layer Chicken Types. Poult. Sci..

[49] Perella F., Mugnai C., Dal Bosco A., Sirri F., Cestola E., Castellini C. (2010). Faba Bean (*Vicia faba* var. minor) as a Protein Source for Organic Chickens: Performance and Carcass Characteristics. Ital. J. Anim. Sci..

[50] Giersberg M.F., Kemper N. (2018). Rearing Male Layer Chickens: A German Perspective. Agriculture.

